# *Antrodia camphorata* Supplementation during Early Life Alters Gut Microbiota and Inhibits Young-Onset Intestinal Tumorigenesis in *APC^1638N^* Mice Later in Life

**DOI:** 10.3390/nu16152408

**Published:** 2024-07-25

**Authors:** Tingchun Lin, Lauren Daddi, Ying Tang, Yanjiao Zhou, Buping Liu, Matthew D. Moore, Zhenhua Liu

**Affiliations:** 1 Department of Nutrition, School of Public Health and Health Sciences, University of Massachusetts, Amherst, MA 01003, USA; tingchunlin@umass.edu (T.L.); yingtang@umass.edu (Y.T.); bupingliu@umass.edu (B.L.); 2 Department of Medicine, University of Connecticut Health Center, Farmington, CT 06030, USA; lauren.daddi@uconn.edu (L.D.); yazhou@uchc.edu (Y.Z.); 3 School of Public Health and Management, Guangzhou University of Chinese Medicine, Guangzhou 510006, China; 4 Department of Food Science, University of Massachusetts Amherst, Amherst, MA 01003, USA; mdmoore@foodsci.umass.edu; 5 UMass Cancer Center, University of Massachusetts Chan Medical School, Worcester, MA 01655, USA

**Keywords:** *Antrodia camphorate*, young-onset colorectal cancer, early-life nutrition, microbiota, Wnt/β-catenin signaling, IGF-1/MAPK/ERK signaling

## Abstract

Young-onset colorectal cancer is an increasing concern worldwide due to the growing prevalence of Westernized lifestyles in childhood and adolescence. Environmental factors during early life, particularly early-life nutrition, significantly contribute to the increasing incidence. Recently, there have been reports of beneficial effects, including anti-inflammation and anti-cancer, of a unique fungus (*Antrodia camphorate*, AC) native to Taiwan. The objective of this study is to investigate the impact of AC supplementation in early life on the development of young-onset intestinal tumorigenesis. *APC^1638N^* mice were fed with a high-fat diet (HF) at 4–12 weeks of age, which is equivalent to human childhood/adolescence, before switching to a normal maintenance diet for an additional 12 weeks up to 24 weeks of age, which is equivalent to young to middle adulthood in humans. Our results showed that the body weight in the HF groups significantly increased after 8 weeks of feeding (*p* < 0.05). Following a switch to a normal maintenance diet, the change in body weight persisted. AC supplementation significantly suppressed tumor incidence and multiplicity in females (*p* < 0.05) and reduced IGF-1 and Wnt/β-catenin signaling (*p* < 0.05). Moreover, it altered the gut microbiota, suppressed inflammatory responses, and created a microenvironment towards suppressing tumorigenesis later in life.

## 1. Introduction

Colorectal cancer (CRC) is a prevalent cancer worldwide [1], with incidence rates declining among adults aged 50 and above but steadily increasing among younger adults [2]. Coinciding with this trend is a significant rise in youth obesity rates [3,4]. Several cohort studies have linked childhood obesity to an increased incidence of CRC in young adults (young-onset CRC) [5,6,7,8]. In addition, lifestyle factors such as a diet high in fat, sugar, and calories and physical inactivity during childhood and adolescence are associated with an increased risk of CRC in adulthood [9,10,11].

Birth cohort effects suggested that early-life risk factors such as obesity might be carried forward to a later life, leading to an upward trend in the incidence of CRC among young adults in recent decades [6,12]. Although observational evidence shows that early-life obesity induced by diet is closely related to young-onset CRC, the underlying mechanisms remain largely undefined. In particular, as the early-life period is critical for major physiological and metabolic changes, exposures to risk factors during this period may profoundly affect physiological integrity and well-developed body function, leading to increased susceptibility to tumorigenesis. Therefore, understanding the etiology and pathophysiological mechanisms behind the association between early-life obesity and young-onset CRC is urgent to relieve the public health burden raised by increased CRC incidence in young adults.

Chemotherapy is the primary strategy for treating CRC, in addition to surgical therapy. However, it is often associated with side effects. Consequently, the development of complementary and alternative medicine, especially the use of natural products as potential anti-cancer remedies, is urgent. *Antrodia camphorate* (AC) is a rare medicinal fungus frequently used in Chinese traditional medicine [13]. Recent studies have shown that AC can suppress proliferation, metastasis, epithelial-to-mesenchymal transition, and induce apoptosis in human CRC cells [14,15,16,17]. Furthermore, AC has demonstrated anti-obesogenic and anti-inflammatory activity in vivo [18,19]. Therefore, AC could be a promising natural product for the prevention and treatment of CRC.

It is widely recognized that obesity is a significant risk factor for CRC in adults [20]. However, little research has been conducted on the potential use of AC to reduce the elevated risk of CRC associated with obesity resulting from a Western lifestyle, particularly the increasing risk of young-onset CRC. Therefore, in the present study, we fed *APC^1638N^* mice a high-fat diet (HF) during their early lives, equivalent to childhood and adolescence in humans, and examined whether AC supplementation could restrain the impact of early-life obesity on intestinal tumorigenesis later in life.

## 2. Materials and Methods

### 2.1. Materials and Reagents

*Antrodia camphorate* supplementation powder (freeze-dried powder of concentrated supernatant collected from submerged fermentation of AC mycelia and containing 44% polysaccharide) was obtained from New Bellus Enterprises Co., Ltd. (Tainan, Taiwan). Gibco^TM^ phosphate buffered saline, TRIzol^®^ reagent, Applied Biosystems^TM^ cDNA Reverse Transcription Kit, SYBR^TM^ Green Master Mix, Pierce^TM^ BCA Protein Assay Kit, and Pierce^TM^ Protease and Phosphatase Inhibitor were purchased from Thermo Fisher Scientific Co. (Waltham, MA, USA). RIPA lysis buffer and bovine serum albumin (BSA) were purchased from MilliporeSigma (Burlington, MA, USA). U-PLEX Mouse Multiplex Immunoassay Kits were purchased from Meso Scale Diagnostics (Rockville, MD, USA). DNeasy^®^ PowerSoil^®^ Pro Kit was purchased from Qiagen Inc. (Germantown, MD, USA). Antibodies against β-catenin (active), p-Akt (Ser473), p-Mek (Ser221), p-Erk1/2 (Thr202/Tyr204), p-GSK-3β (Ser9), GAPDH, and anti-rabbit IgG and horseradish peroxidase (HRP)-linked antibodies were purchased from Cell Signaling Technology (Beverly, MA, USA).

### 2.2. Animal Study

The Institutional Animal Care and Use Committee of the University of Massachusetts, Amherst, approved the animal use protocol (protocol number: 930), and all animal experiments were conducted in compliance with the U.S. Animal Welfare Act and the Guide for the Care and Use of Laboratory Animals. This study was also carried out in compliance with the ARRIVE guidelines [21]. To investigate the effects of early-life nutrition on young-onset colorectal tumorigenesis, we selected the *Apc^1638N^* mouse model, which carries a mutation at codon 1638 of the *Apc* gene that is responsible for familial adenomatous polyposis (FAP), a predisposition syndrome to colorectal tumor [22]. Compared to *Apc^min/+^*, which is commonly used in the research of colorectal cancer, *Apc^1638N^* has a longer lifespan of a year or more and develops attenuated polyposis with fewer than 100 polyps. These genetic and physiological differences make *Apc^1638N^* a more suitable model to study the interaction of environmental factors, such as dietary effects, with an inherited mutation of the *Apc* gene [22].

**Figure 1:**
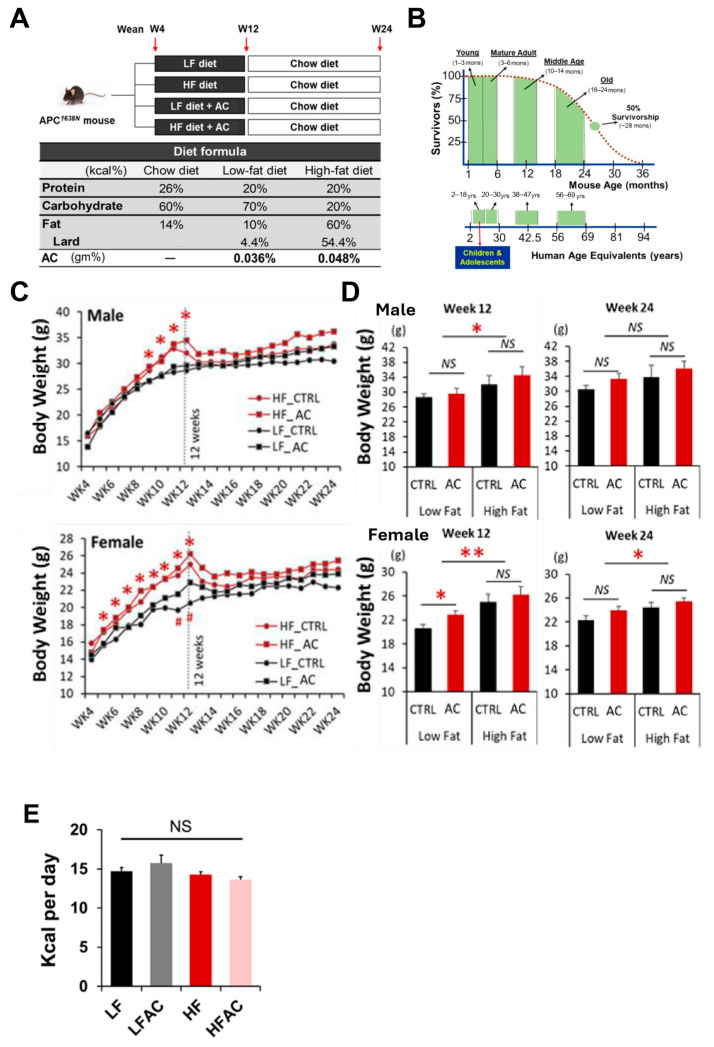
Experimental design and mouse body weight changes during the feeding period. (**A**) Experimental design. The *APC^1638N^* model was used to study intestinal tumorigenesis. Mice were randomly divided into low-fat diet (LF) with and without AC supplementation and high-fat diet (HF) with and without AC supplementation groups during weeks 4 to 12, and then all groups switched back to a normal maintenance diet for another 12 weeks. (**B**) Mouse and human life phase equivalencies. The experimental diet feeding period is equivalent to childhood and adolescence in humans. (**C**) Growth curves for 20-week feeding experiments in male and female mice. (**D**) Body weight comparisons among the LF group with and without AC supplementation and HF group with and without AC supplementation on weeks 12 and 24. (**E**) Feed intake comparisons among the LF group with and without AC supplementation and the HF group with and without AC supplementation groups during the initial 8-week period. Data are presented as mean ± SEM, n = 10–13 for each group. ^#^ *p* < 0.1, * *p* < 0.05, ** *p* < 0.01. APC: adenomatous polyposis coli. AC: *Antrodia camphorata* supplementation (powder). *NS*: not statistical significance.

Our study aimed to determine the influence of early-life nutrition on young-onset colorectal tumorigenesis and to explore whether the hypothetical colorectal tumorigenesis pathways, including metabolic dysregulation, chronic inflammation, and microbial dysbiosis, provoked by early-life, dietary-induced obesity can be impeded, at least in part, by a dietary anti-tumor approach via complementing AC supplementation in the mouse diet. To achieve this, we fed *Apc^1638N^* mice a HF (60% calories from fat) or LF diet (control diet, 10% calories from fat) (D12492 and D12450B, Research Diets, Inc.; see Supplementary Table S1 for detailed ingredients) ad libitum starting at 4 weeks of age for 8 weeks to reflect early-life nutrition in humans (Figure 1A,B). Subsequently, we switched all groups back to a standard chow diet (14% calories from fat) (LabDiet #5P76, Land O’Lakes, Inc., Arden Hills, MN, USA) for an additional 12 weeks [23]. Additionally, the supplemental groups were given AC supplementation at a dose of 90 mg/kg for the first 8 weeks of the feeding experiment (Figure 1A) [18].

### 2.3. Insulin Level and Pro-Inflammatory Cytokine Profiling

Plasma insulin levels and pro-inflammatory mediators in plasma and the intestine were measured using multiplex immunoassays based on electrochemiluminescence with the Meso Scale Discovery System^®^ (Meso Scale Diagnostics, Rockville, MD, USA). The assays were performed according to the manufacturer’s instructions. In brief, antibodies against insulin, TNF-α, IL-6, IL-17A, and CCL-2 were coated on a 96-well multiplex assay plate. Calibrator standards or prepared samples (50 µL) were added to each well, followed by incubation at room temperature for 2 h. The detection antibody solution (50 µL) was then added to each well, and the plate was incubated at room temperature for 1 h. To analyze the expression of each targeted protein, 150 µL of MSD GOLD read buffer B was added to each well, and the plate was analyzed using the MSD MESO QuickPlex SQ 120 (Meso Scale Diagnostics, Rockville, MD, USA). A four-parameter logistic fit curve was generated for each analyte using the standards, and the levels of insulin and pro-inflammatory mediators in the samples were calculated accordingly. All target proteins were expressed as pg per milliliter, and all standards and samples were measured in duplicate. To account for differences in protein concentration between samples, the protein expressions in the intestinal samples were normalized by dividing the measured protein levels by the total protein content of each sample. The total protein content was determined using the Pierce^TM^ BCA Protein Assay Kit (Thermo Fisher Scientific Co., Waltham, MA, USA), according to the manufacturer’s instructions. Insulin resistance was assessed using the HOMA-IR index, which was calculated as [fasting serum glucose (mg/dL) × fasting serum insulin (μIU/mL)/405].

### 2.4. Real-Time PCR

Total RNAs were extracted from tissues using TRIzol^®^ reagent (Invitrogen™, Carlsbad, CA, USA) as per the manufacturer’s instructions and stored at −80 °C. The concentration and purity of RNA samples were determined using a NanoDrop™ Lite Spectrophotometer (Thermo Scientific™, Waltham, MA, USA). First-strand cDNAs were synthesized from RNA samples using a high-capacity cDNA reverse transcription kit (Applied Biosystems™, Carlsbad, CA, USA). The relative expression of target genes was measured using SYBR^TM^ Green Master Mix (Applied Biosystems™, Carlsbad, CA, USA) and ViiA™ 7 Real-Time PCR System (Applied Biosystems^®^, Carlsbad, CA, USA) with the following conditions: hold stage starting at 50 °C for 2 min, 95 °C for 10 min, 40 cycles starting at 95 °C for 15 s, 60 °C for 1 min, melt curve stage starting at 95 °C for 15 s, 60 °C for 1 min, and 95 °C for 15 s. The primers for amplifying the target genes (*Igf1*, *Igf1r*, *Akt*, *Hdac6*, *c-Jun*, *c-Myc*, *Ccnd1*, *Axin2*, *Tnf*, *Il1β*, *Il6*, *Il17A*, *Ccl2*, *Ptgs2*, *Tgfb1*, and *Gapdh*) were designed using PrimerBank (https://pga.mgh.harvard.edu/primerbank/, accessed on 2 March 2022). The primers used are listed in Supplementary Table S2. The gene expression was normalized to the housekeeping gene *Gapdh* (ΔCt = Ct-_target gene_ − Ct-*_Gapdh_*). Statistical analyses were performed based on ΔCt, and relative expression is reported as 2^−ΔΔCt^, where ΔΔCt = ΔCt-_experiment_ − ΔCt-_control_.

### 2.5. Immunoblotting

Total protein was extracted from tissues using RIPA lysis buffer (MilliporeSigma, Burlington, MA, USA) and stored at −80 °C. The total protein content was determined using the Pierce^TM^ BCA Protein Assay Kit (Thermo Fisher Scientific Co., Waltham, MA, USA), according to the manufacturer’s instructions. Western blot analysis was performed to analyze protein expression as previously described [24]. Briefly, 20 μg of protein from each sample was loaded into a 10% SDS-PAGE gel for protein separation and then blotted onto an Immun-Blot^®^ PVDF Membrane (Bio-Rad, Hercules, CA, USA). After blocking the membranes in 5% BSA (Sigma-Aldrich, St. Louis, MO, USA) in Tris Buffered Saline with Tween 20 (TBST) for 1 h, they were incubated overnight at 4 °C with primary antibodies against β-catenin (1:1000), p-Akt (1:1000), p-Mek (1:1000), p-Erk1/2 (1:1000), p-Gsk3β (1:1000), and GAPDH (1:10,000), followed by incubation with HRP-conjugated secondary antibody (1:5000) for 1 h at room temperature. Antibody binding was detected by incubating the membranes with enhanced chemiluminescence substrate (Bio-Rad, Hercules, CA, USA), and images were captured using the Odyssey^®^ Fc Imaging System (LI-COR Biosciences, Lincoln, NE, USA). Relative protein expression was quantified using Image J software (v1.53v) after normalizing to the corresponding loading control.

### 2.6. Microbiota Analysis

Cecal contents were collected during dissection, snap-frozen in liquid nitrogen, and stored at –80 °C. Metagenomic DNA from cecal contents was extracted using the DNeasy^®^ PowerSoil^®^ Pro Kit (Qiagen Inc., Germantown, MD, USA) according to the manufacturer’s protocol and stored at –80 °C. The concentration and purity of DNA samples were determined using a NanoDrop™ Lite Spectrophotometer (Thermo Scientific™, Waltham, MA, USA). 16S rRNA gene amplification and sequencing were performed to analyze the gut microbiota as previously described [25]. Briefly, 30 ng of extracted DNA from each sample was used for PCR amplification of the V4 region of the 16S rRNA gene using the forward primer 515F and reverse primer 806R with Illumina adapters and dual indices (eight base pairs). The PCR condition comprised pre-denaturation at 95 °C for 3.5 min, followed by 30 cycles starting at 95.0 °C for 30 s, 50.0 °C for 30 s, and 72 °C for 90 s, and a final extension of 72 °C for 10 min. Amplicons were identified and quantified using the QIAxcel DNA Fast Analysis (Qiagen Inc., Germantown, MD, USA). Polymerase chain reaction (PCR) products were normalized based on the concentration of DNA from 250–400 bp and purified using Mag-Bind^®^ TotalPure NGS (Omega Bio-tek, Inc., Norcross, GA, USA) according to the manufacturer’s instructions, followed by sequencing on the Illumina MiSeq System (Illumina Inc., San Diego, CA, USA).

The 16S rRNA gene sequences were analyzed using the BaseSpace software (v7.7.0) (Illumina, San Diego, CA, USA), DADA2 data processing pipeline, Phyloseq R package, and final taxonomic assignment was conducted using RDP-classifier (v2.11) (Michigan State University, East Lansing, MI, USA) as previously described [25]. To determine species richness and diversity within each sample, α-diversity was calculated using the Shannon diversity and Richness metrics in RStudio version 2022.12.0 (RStudio, Boston, MA, USA) with the ggplot2 R package. Differences between groups were identified using the Wilcoxon test. To determine differences in taxonomic profiles between samples, β-diversity was analyzed using PCoA with Bray–Curtis dissimilarity metrics in RStudio version 2022.12.0 (RStudio, Boston, MA, USA) with the vegan R package. Permutational multivariate analysis of variance on Bray–Curtis dissimilarity and Jaccard indices was conducted to determine the difference between the two groups. The effect of treatments on the relative abundance of taxa was determined using LEfSe with Galaxy (https://huttenhower.sph.harvard.edu/galaxy/, accessed on 30 December 2022) [26]. The effect size of differentially represented taxa was represented by LDA scores, and an adjusted *p*-value < 0.05 by false discovery rate was considered statistically significant.

### 2.7. Statistical Analyses

Data are presented as means ± SEM. Fisher’s exact test was used to identify the difference between two groups for tumor incidence analysis, and a two-tailed, unpaired t-test was used for numerical data analysis, unless stated otherwise. The correlation matrix was carried out using RStudio version 2022.12.0 (RStudio, Boston, MA, USA) with the corrplot and Hmisc R packages. Statistical analyses were performed using SAS program version 9.4 (SAS Institute, Cary, NC, USA), GraphPad Prism version 9 (GraphPad Software, San Diego, CA, USA), and RStudio version 2022.12.0 (RStudio, Boston, MA, USA).

## 3. Results

### 3.1. HF Diet in Early Life Increased Body Weight and Interfered with Glucose Homeostasis in Female APC^1638N^ Mice, Whereas AC Supplementation Attenuated the Effect

**Figure 2:**
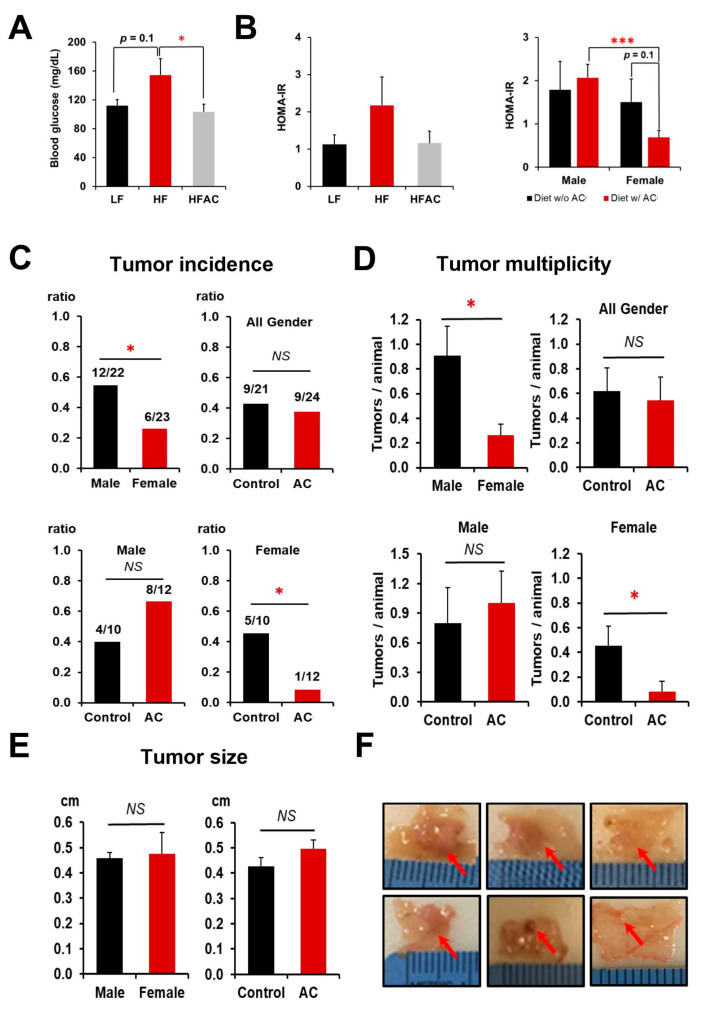
The influence of a high-fat diet and AC supplementation in early life on blood glucose homeostasis and intestinal tumorigenesis in *APC^1638N^* mice. (**A**) The effects of an early-life high-fat diet (HF) with and without AC supplementation on blood glucose changes. (**B**) The effects of an early-life HF diet with and without AC supplementation on insulin resistance and the effects of AC supplementation on insulin resistance in male and female mice. (**C**) Tumor incidence in male and female mice and the differentiated impacts of AC supplementation in males and females. (**D**) Tumor multiplicity in male and female mice and the differentiated impacts of AC supplementation in males and females. Tumor multiplicity was calculated based on the number of tumors divided by the number of mice in each group. (**E**) Tumor size between male and female mice and the impact of AC supplementation on tumor size. (**F**) Representative pictures of intestinal tumors. Representative tumor pictures from a mouse fed without AC supplementation (**top**) and representative tumor pictures from a mouse fed with AC supplementation (**bottom**). Red arrows indicate the tumors. Control and AC in figure (**C**–**E**) meant experimental diets without and with AC supplementation, regardless of whether fed a LF or HF diet. Data are presented as mean ± SEM. * *p* < 0.05. *** *p* < 0.001. *NS*: not statistical significance. w/: with. w/o: without.

After being fed a HF diet for 8 weeks, both male and female mice showed a significant increase in body weight compared to the low-fat diet (LF) group (*p* < 0.05, Figure 1C,D), with an apparent larger magnitude for female mice, which is out of our expectation. There was no significant difference in daily energy intake between groups (Figure 1E). However, the addition of AC supplementation to the experimental diet did not have a significant effect on body weight change (Figure 1C,D). Following an additional 12 weeks of normal chow diet feeding, the body weight increase in the HF groups remained but was less pronounced, particularly in the female mice (*p* < 0.05, Figure 1D). While AC supplementation into both LF and HF groups during early life did not affect body weight gain over the entire experimental period, it significantly reduced blood glucose levels, which were otherwise increased by a HF feeding (*p* < 0.05, Figure 2A). Furthermore, it was apparent that the elevated homeostatic model assessment for insulin resistance (HOMA-IR) index caused by HF feeding in early life showed a decreasing trend with AC supplementation, although the differences did not reach statistical significance. Moreover, similar trends were observed in both male and female mice, with the effect being particularly evident in female mice (Figure 2B). The impacts of AC supplementation on glucose and HOMA-IR in LF groups were not significant.

### 3.2. Antrodia camphorate Supplementation during Early Life Inhibited Intestinal Tumorigenesis in Female APC^1638N^ Mice Later in Life

The results showed that early-life consumption of a HF diet increased the incidence of intestinal tumors in both male and female *APC^1638N^* mice compared to those fed a LF diet, with tumor incidences of 48% and 32%, respectively (*p* < 0.10). However, the addition of AC to the diet during early life significantly suppressed tumor incidence (50% vs. 8%) and multiplicity (0.455 ± 0.157 vs. 0.083 ± 0.083) only in female *APC^1638N^* mice, regardless of whether they were fed a LF or HF diet (*p* < 0.05, Figure 2C,D,F), although no effects were observed on tumor size (Figure 2E,F). When comparing intestinal tumorigenesis between genders, both tumor incidence and tumor multiplicity were significantly higher in males than in females, with a tumor incidence of 55% for males and 26% for females and a tumor multiplicity of 0.909 ± 0.236 for males and 0.261 ± 0.094 for females (*p* < 0.05, Figure 2C,D).

### 3.3. HF Diet in Early Life Induced the Activation of IGF-1/MAPK Signaling in APC^1638N^ Mice, Whereas AC Supplementation Attenuated This Effect

**Figure 3:**
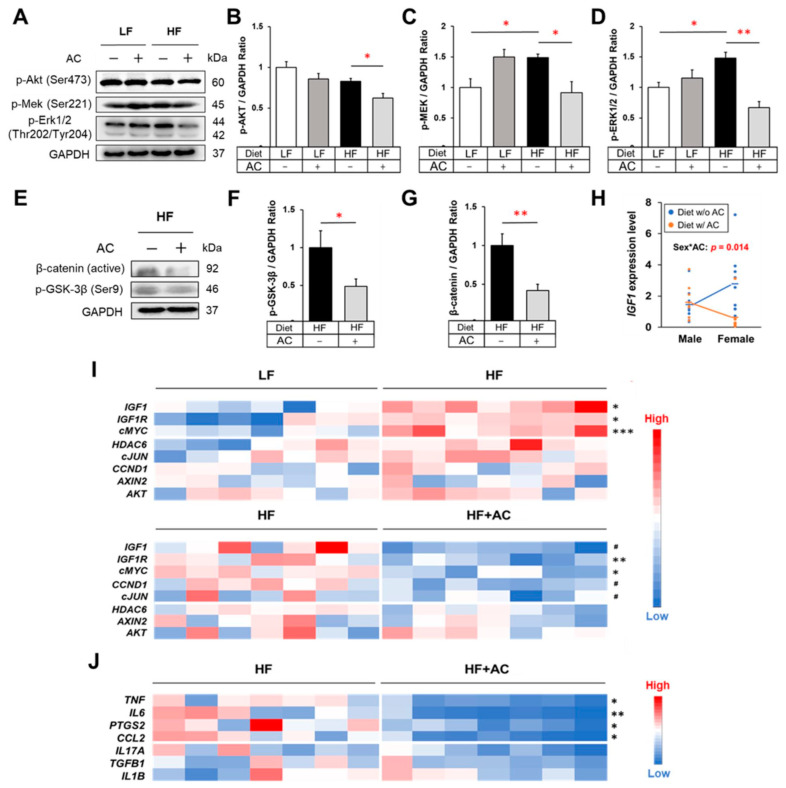
The influence of high-fat diet and AC supplementation in early life on IGF-1/MAPK signaling, Wnt/β-catenin signaling, and inflammatory cytokines in *APC^1638N^* mice. (**A**) Western blots for phospho-Akt, phospho-Mek, and phospho-Erk in the intestine. (**B**–**D**) The effects of early-life high-fat diet (HF) and AC supplementation on protein expression of phospho-Akt, phospho-Mek, and phospho-Erk in the intestine. (**E**) Western blots for phospho-GSK-3β and active β-catenin in the intestine. (**F**,**G**) The effects of AC supplementation in early life on protein expression of phospho-GSK-3β and β-catenin in the intestine of mice fed with an early-life HF diet. (**H**) Test of the interaction between sex and AC supplementation on *Igf1* gene expression. (**I**) Gene expression heatmaps of IGF-1/MAPK signaling and Wnt/β-catenin signaling-related genes in the small intestine. (**J**) Gene expression heatmap for inflammatory mediators in the small intestine. Data are presented as mean ± SEM, n = 8–11 for each group. ^#^ *p* < 0.1, * *p* < 0.05, ** *p* < 0.01, *** *p* < 0.001. w/: with. w/o: without. Original blots are presented in Supplementary Figures S2–S6.

Insulin resistance is considered a risk factor contributing to the development of CRC. To investigate this, we measured the activation of tumorigenic insulin-like growth factor-1 (IGF-1) signaling, which is related to insulin dysfunction [27,28]. Our results indicated that a HF diet in early life significantly elevated both *Igf1* and *Igf1r* expression in the intestine and increased the expression of downstream genes in the IGF-1/MAPK/ERK signaling pathway (*p* < 0.05, Figure 3A–D,I). However, supplementation with AC during early life significantly inhibited the stimulating effect caused by an early-life HF diet on the expression of p-Mek and p-Erk1/2 (*p* < 0.05 and *p* < 0.01, respectively, Figure 3A–D). It also significantly suppressed *Igf1r* expression (*p* < 0.01, Figure 3I).

Additionally, to characterize the varying effects of AC supplementation during early life on the declining intestinal tumor incidence between male and female mice, we assessed the interaction between sex and AC supplementation on gene expression. Both the diet with and without AC comprised individuals from both the LF and HF groups. The results showed that *Igf1* expression was differentially affected by AC supplementation depending on gender, with reduced *Igf1* expression attributed to AC supplementation observed only in female mice (Sex*AC: *p* = 0.014, Figure 3H). Statistical analysis also confirms a significant test of interaction denoted by Sex*AC treatment in the HF group (*p* = 0.037). This observed pattern in the HF group further reinforces the interaction between sex and AC treatment regarding *Igf1* gene expression.

### 3.4. Antrodia camphorate Supplementation during Early Life Mitigated Inflammatory Response and Decreased Wnt/β-Catenin Signaling in APC^1638N^ Mice Later in Life

Studies have shown that chronic inflammation is a significant risk factor for diet-induced CRC, and the persistent activation of Wnt/β-catenin signaling due to inflammation is a critical tumorigenic phenomenon in the development of CRC [29,30,31,32]. As shown in Figure 3J, AC supplementation during early life significantly attenuated the gene expression of pro-inflammatory mediators, including *Tnf*, *Il6*, *Ptgs2*, and *Ccl2*, in the intestine of *APC^1638N^* mice (*p* < 0.05). Moreover, the intestinal protein levels of TNF-α, IL-6, and IL-17A were decreased in both male and female mice that received AC supplementation, regardless of whether they were fed a HF or LF diet (*p* < 0.05, *p* < 0.05, and *p* < 0.1, respectively, Supplementary Tables S4 and S5). Although other changes were noted, a decrease in circulating pro-inflammatory mediators was not observed (Supplementary Table S3). Simultaneously, AC supplementation significantly suppressed the expression of p-GSK-3β and active β-catenin in the intestine of early-life HF-fed mice (*p* < 0.05 and *p* < 0.01, respectively, Figure 3E–G).

### 3.5. Antrodia camphorate Supplementation during Early Life Altered the Diversity and Composition of the Gut Microbiota in APC^1638N^ Mice

**Figure 4:**
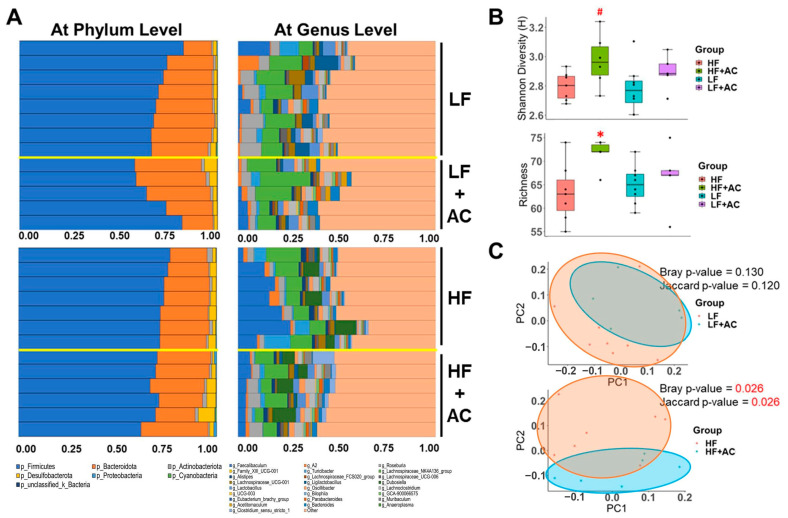
The influence of a high-fat diet and AC supplementation in early life on cecal microbial composition in *APC^1638N^* mice. (**A**) Bar plots of relative abundance of microbiota at phylum level and at genus level for the comparisons between low-fat diet (LF) with and without AC supplementation and high-fat diet (HF) with and without AC supplementation. (**B**) Shannon and Richness metrics were used to determine α-diversity. (**C**) Principal Coordinates Analysis (PCoA) plot illustrates β-diversity. Red text indicates a significant difference. Permutational multivariate analysis of variance (PERMANOVA) in Jaccard and Bray–Curtis metrics indicates the statistically significant differences between an early-life HF diet with and without AC supplementation. N = 5–8 for each group. ^#^ *p* < 0.1, * *p* < 0.05.

To explore the potential impact of an early-life HF diet and AC supplementation on the gut microbiota of *APC^1638N^* mice later in life, we conducted an analysis of the cecal microbiota composition. Our results revealed that the cecal microbiota of mice fed with HF and LF diets were primarily composed of the phyla *Firmicutes*, *Bacteroidota*, *Actinobacteriota*, and *Desulfobacterota* (Figure 4A). However, there was no statistically significant difference in α diversity, as determined by Shannon and Richness metrics (Figure 4B), or the *Firmicutes*/*Bacteroidota* (F/B) ratio between the early-life HF and LF groups, whereas the Principal Coordinates Analysis (PCoA) indicated that HF group had a different taxonomic profile compared to the LF group (Bray *p*-value = 0.001, Jaccard *p*-value = 0.002), with higher abundance in *Bacteroidota* (genus *Prevotellaceae UCG 001*), and *Firmicutes* (genus *Anaeroplasma*, *Ileibacterium*, *Lactobacillus*, *Lachnospiraceae UCG 001*, *Roseburia*, *Monoglobus*, and *Anaerotruncus*) in the LF group and higher abundance in *Desulfobacterota* (genus *Bilophila*), *Bacteroidota* (genus *Alistipes*), and *Firmicutes* (genus *Faecalibaculum*, and *Lachnospiraceae GCA 900066575*) in the HF group.

**Figure 5:**
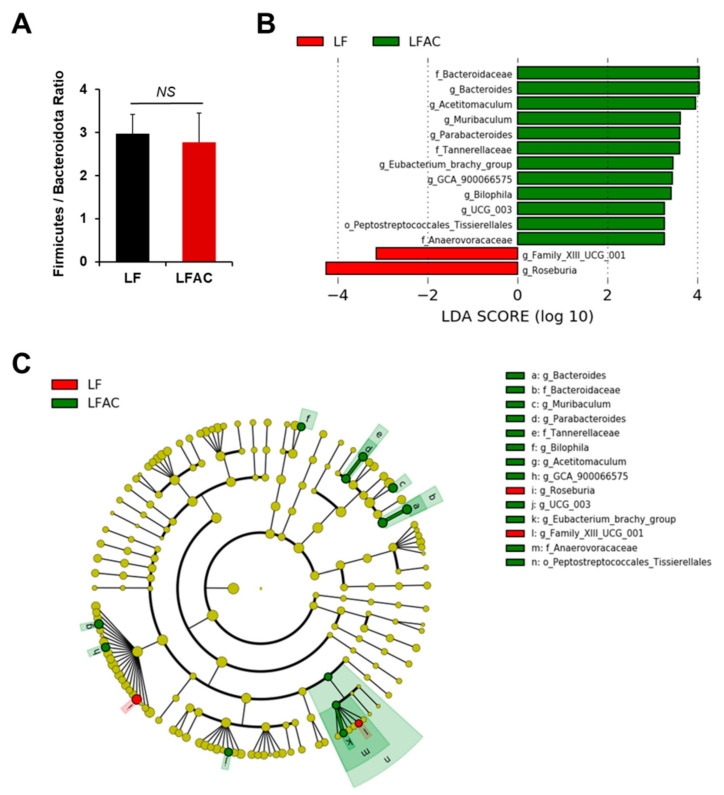
The influence of AC supplementation in early life on cecal microbial composition in *APC^1638N^* mice fed with a low-fat diet. (**A**) *Firmicutes*/*Bacteroidota* ratio; (**B**) Linear discriminant analysis (LDA) effect size (LEfSe) for mice fed with a low-fat diet (LF) with and without AC supplementation shows differential microbiota at each taxon level. The threshold on the LDA score for discriminative features was set to 2.0. The abundance of the listed taxon of bacteria was significantly different (*p* < 0.05) between groups. Green bars indicate a higher abundance of taxa in the LF group with AC supplementation, and red bars indicate a higher abundance of taxa in the LF group without AC supplementation. (**C**) Cladogram of the LEfSe analysis between groups. The microbial composition was compared at different taxon levels. The taxon level is abbreviated as p-phylum, c-class, o-order, f-family, and g-genus. *NS*: not statistical significance.

The AC supplementation did not alter the microbial diversity in early-life LF-fed mice (Figure 4A–C and Figure 5A). Nevertheless, it significantly increased the abundance of *Bacteroidota* (genus *Bacteroides*, *Muribaculum*, and *Parabacteroides*, LDA score of 4.04, 3.63, and 3.61, respectively, *p* < 0.05), *Desulfobacterota* (genus *Bilophila*, LDA score of 3.42, *p* < 0.05), and *Firmicutes* (genus *Acetitomaculum*, *Lachnospiraceae GCA 900066575*, *Oscillospiraceae UCG 003*, and *Eubacterium brachy group*, LDA score of 3.97, 3.45, 3.27, and 3.47, respectively, *p* < 0.05) in LF-fed mice, as shown by the Linear Discriminant Analysis Effect Size (LEfSe) test (Figure 5B,C).

**Figure 6:**
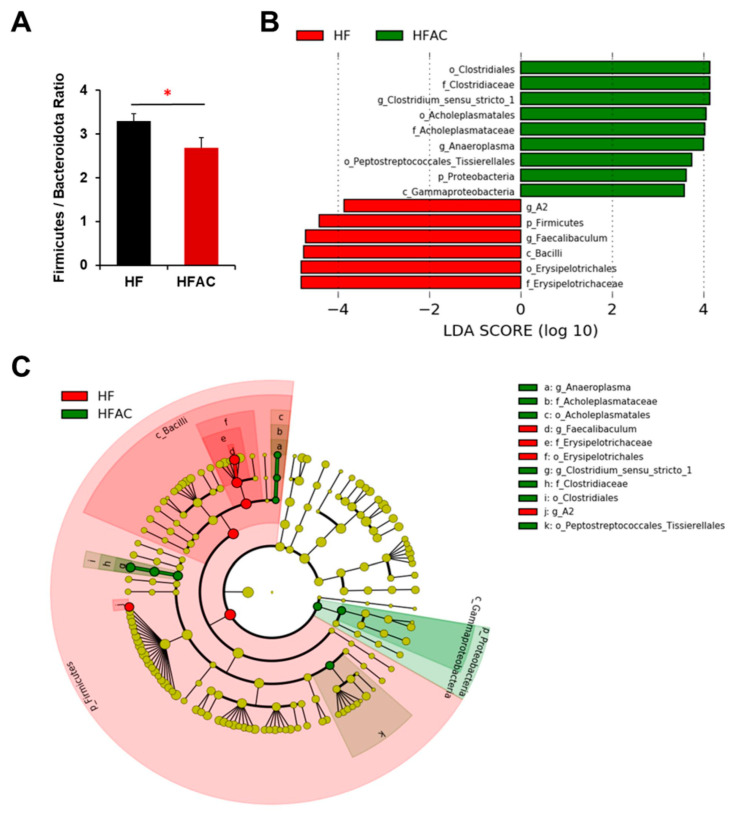
The influence of AC supplementation in early life on cecal microbial composition in *APC^1638N^* mice fed with an early-life high-fat diet. (**A**) *Firmicutes*/*Bacteroidota* ratio (**B**) Linear discriminant analysis (LDA) effect size (LEfSe) for mice fed with a high-fat diet (HF) with and without AC supplementation shows differential microbiota at each taxon level. The threshold on the LDA score for discriminative features was set to 2.0. The abundance of the listed taxon of bacteria was significantly different (*p* < 0.05) between groups. Green bars indicate a higher abundance of taxa in HF group with AC supplementation, and red bars indicate a higher abundance of taxa in HF group without AC supplementation. (**C**) Cladogram of the LEfSe analysis between groups. The microbial composition was compared at different taxon levels. The taxon level is abbreviated as p-phylum, c-class, o-order, f-family, and g-genus. * *p* < 0.05.

However, the addition of AC supplementation significantly increased α diversity and decreased the F/B ratio in early-life HF-fed mice (*p* < 0.05, Figure 4B and Figure 6A). The taxonomic profile was notably altered in early-life HF-fed mice complemented with AC supplementation, as shown by the PCoA (Bray *p*-value = 0.026, Jaccard *p*-value = 0.026, Figure 4C). Specifically, *Firmicutes* (genus *Lachnospiraceae A2* and *Faecalibaculum*, LDA score of 3.86 and 4.70, respectively, *p* < 0.05) were more abundant in HF-fed mice, whereas *Firmicutes* (genus *Anaeroplasma* and *Clostridium sensu stricto 1*, LDA score of 4.00 and 4.14, respectively, *p* < 0.05) and *Proteobacteria* (class *Gammaproteobacteria*, LDA score of 3.59, *p* < 0.05) were more abundant once complemented with AC supplementation (Figure 6B,C).

### 3.6. Antrodia camphorate Supplementation during Early Life Altered the Abundance of Gut Bacteria That Was Linked with Increased Igf1 Expression and Improved Inflammatory Status in APC^1638N^ Mice Later in Life

**Figure 7:**
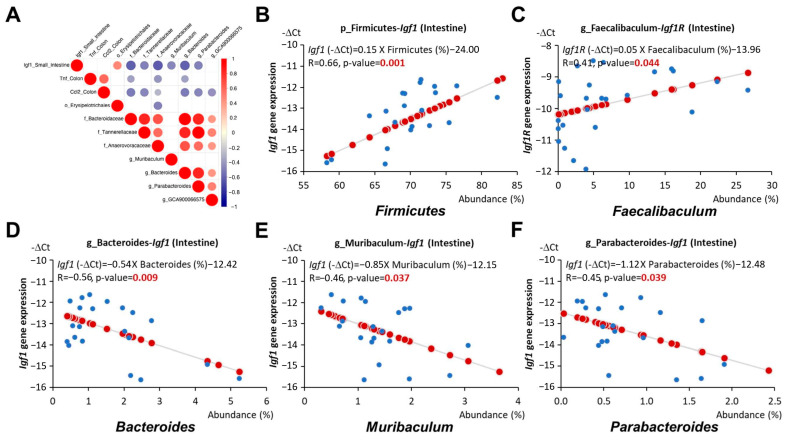
Relationship between gut microbiota and *Igf1* expression in *APC^1638N^* mice. (**A**) Correlation matrix of gut microbiota and *Igf1* expression in the small intestine or cytokines *Tnf* and *Ccl2* expressions in the colon. Red or blue dots represent significantly positive or negative coefficients. (**B**) Linear relationships between *Firmicutes* and *Igf1* expression in the small intestine. (**C**) Linear relationships between *Faecalibaculum* and *Igf1* expression in the small intestine. (**D**) Linear relationships between *Bacteroides* and *Igf1* expression in the small intestine. (**E**) Linear relationships between *Muribaculum* and *Igf1* expression in the small intestine. (**F**) Linear relationships between *Parabacteroides* and *Igf1* expression in the small intestine. Blue circles indicate the representative -∆Ct value and abundance (%) of each mouse. Red circles indicate the linear relationships between the x and y axes.

The correlation analysis conducted between the gut microbiota and all biomarkers measured in our study indicated a positive correlation between the phylum *Firmicutes* and the genus *Faecalibaculum* with *Igf1* and *Igf1r* expression in the intestine, respectively (*p* = 0.001 and 0.044, respectively, Figure 7B,C). The abundance of these bacteria was significantly increased in early-life HF-fed mice but decreased with the addition of AC supplementation (Figure 6B,C). In contrast, the family *Anaerovoracaceae* and the genera *Bacteroides*, *Muribaculum*, and *Parabacteroides* showed a negative association with *Igf1* expression in the intestine (*p* = 0.003, 0.009, 0.037, and 0.039, respectively, Figure 7A,D–F). The abundance of these bacteria was elevated in mice fed an early-life LF diet complemented with AC supplementation (Figure 5B,C). The comprehensive correlation matrix is included in Supplementary Figure S1.

**Figure 8:**
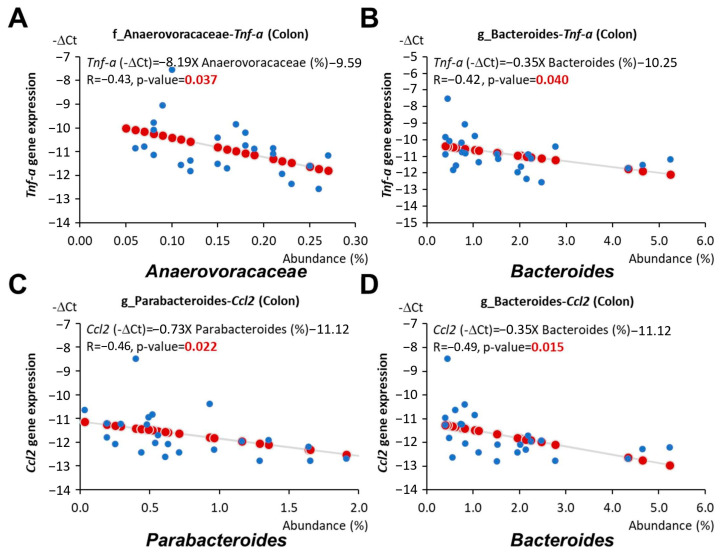
Relationship between gut microbiota and cytokine gene expressions in young adulthood *APC^1638N^* mice. (**A**) Linear relationships between *Anaerovoraceae* and *Tnf* expression in the colon. (**B**) Linear relationships between *Bacteroides* and *Tnf* expression in the colon. (**C**) Linear relationships between *Parabacteroides* and *Ccl2* expression in the colon. (**D**) Linear relationships between *Bacteroides* and *Ccl2* expression in the colon. Blue circles indicate the representative -∆Ct value and abundance (%) of each mouse. Red circles indicate the linear relationships between the x and y axes.

Furthermore, the abundance of the family *Anaerovoracaceae* and the genus *Bacteroides* was associated with reduced *Tnf* expression in the colon (*p* = 0.037 and 0.04, respectively, Figure 8A,B), and the abundance of the genus *Parabacteroides*, *Bacteroides*, and *Lachnospiraceae GCA 900066575* was linked to reduced colonic *Ccl2* expression (*p* = 0.022, 0.015, and 0.036, respectively, Figure 7A and Figure 8C,D). The abundance of these bacteria was elevated in mice fed an LF diet with AC supplementation (Figure 5B,C).

## 4. Discussion

CRC is the third most common cancer diagnosed in both men and women worldwide [33]. Despite the overall burden of CRC has declined in recent years, largely due to the implementation of CRC screening with colonoscopy, evidence from epidemiological studies suggests that the incidence rates of CRC continue to rise in adults under the age of 50 [2]. Concurrently, rates of obesity among youth have incrementally increased over the past few decades [3,4]. While the link between obesity and CRC in adulthood has been well documented, the connection between early-life obesity and colorectal tumorigenesis later in life, as well as the underlying mechanisms, remain a critical research gap [34,35]. In the present study, we illustrated the impact of an early-life HF diet on intestinal tumorigenesis through insulin dysfunction and microbiota alteration, specifically in female *APC^1638N^* mice. AC supplementation during early life attenuated the adverse effects of a HF diet and reduced the levels of inflammatory mediators and tumorigenic Wnt/β-catenin signaling in the intestine.

As expected, a 60% kcal HF diet caused a significant increase in body weight during their early lives, and the difference persisted after the mice were switched back to a normal chow diet for an additional 12 weeks (Figure 1C,D). The addition of AC during early life did not attenuate the body weight gain caused by a HF diet, whereas it significantly reduced the development of intestinal tumors later in life in female mice (Figure 2C,D). To our surprise, we observed a numerical but not statistically significant increase in tumor development in males. This is most likely attributed to the more significant increase in body weight in males fed with the AC supplementation at the end of the 24-week experiment. Generally, male mice are more responsive to diet-induced obesity, and the C57BL/6 mouse strain has a large variation in response to HF-induced obesity [36], resulting in a numerical but not statistical alteration of tumor development in males. Further research regarding the gender disparity is warranted. Nevertheless, to further understand the gender differences in intestinal tumorigenesis in response to AC supplementation, we explored the differences in biomarkers between genders. Interestingly, we found that AC supplementation during early life attenuated insulin resistance and suppressed *Igf1* expression later in life exclusively in female mice (Figure 2B and Figure 3H). These results suggest that AC supplementation during early life could be more beneficial for relieving the metabolic burden caused by abnormal glucose homeostasis in females than in males. These differentiated results might be attributed to the interaction of estrogen with the effects of AC supplementation and the IGF-1 axis [37,38,39]. Research is needed to further reveal the gender disparity.

Chronic inflammatory status is known to activate Wnt/β-catenin signaling in the intestine, which is a pivotal tumorigenic pathway involved in over 90% of CRC cases, via pro-inflammatory mediators such as TNF-α [40], IL-6 [41], PGE2 [42], and CCL2 [43]. Therefore, the alleviation of intestinal inflammation due to AC supplementation observed in the present study may also explain why the Wnt/β-catenin signaling pathway was attenuated, indicating the potential anti-cancer activity of AC supplementation (Figure 3J and Supplementary Tables S4 and S5).

There is no doubt that the development of CRC is intimately associated with dysbiosis, which can lead to the production of toxic or carcinogenic metabolites and affect host metabolism and immune system function [44]. Research has shown that the composition of the microbiota can be significantly influenced by diet and obesity [45,46,47,48]. Indeed, we observed the different cecal taxonomic profiles in early-life HF and LF groups even after all the groups were switched to a normal diet after 12 weeks. Furthermore, our results showed that AC supplementation in the HF group during early life significantly reduced the F/B ratio, which was reported to be highly associated with obesity and related metabolic disorders [46,49,50,51]. It also significantly altered the microbiota composition in both early-life HF- and LF-fed mice, with a higher abundance of *Anaeroplasma*, *Clostridium sensu stricto 1*, *Gammaproteobacteria*, *Bacteroides*, *Muribaculum*, *Parabacteroides*, *Bilophila*, *Acetitomaculum*, *Lachnospiraceae GCA 900066575*, *Oscillospiraceae UCG 003*, and *Anaerovoracaceae* but a lower abundance of *Lachnospiraceae A2* and *Faecalibaculum* (Figure 5 and Figure 6).

Previous research has shown that certain gut bacteria, including *Anaeroplasma*, *Bacteroides*, *Muribaculum*, *Parabacteroides*, and *Lachnospiraceae GCA 900066575*, are decreased in HF-fed mice or obese individuals, indicating their potential roles in alleviating obesity and metabolic dysfunctions [52,53,54,55,56,57,58,59,60,61,62,63,64]. *Anaeroplasma*, *Clostridium sensu stricto 1*, *Bacteroides*, *Muribaculum*, *Parabacteroides*, and *Anaerovoracaceae* have also been reported to reduce inflammatory responses or modulate the immune system, making them potential anti-inflammatory bacteria [57,58,65,66,67,68,69,70,71]. Additionally, some studies have highlighted that *Clostridium sensu stricto 1*, *Bacteroides*, *Muribaculum*, *Parabacteroides*, *Acetitomaculum*, and *Anaerovoracaceae* are capable of producing short-chain fatty acids (SCFA) with anti-obesity, anti-inflammatory, immunoregulatory, and anti-cancer properties [66,70,72,73,74,75,76,77]. Conversely, *Lachnospiraceae A2* and *Faecalibaculum* have been implicated in inflammatory bowel diseases, insulin resistance, obesity, and chronic inflammation [57,78,79,80,81].

Consistent with previous findings, our results showed that the abundance of *Anaerovoracaceae*, *Bacteroides*, *Muribaculum*, and *Parabacteroides*, which was increased by AC supplementation during early life, was negatively correlated with *Igf1* expression in the intestine (Figure 7A,D–F). The abundance of *Anaerovoracaceae* and *Bacteroides* was also associated with reduced *Tnf* expression in the colon (Figure 8A,B). Moreover, the abundance of *Parabacteroides*, *Bacteroides*, and *Lachnospiraceae GCA 900066575*, which was elevated by AC supplementation during early life, was linked to reduced colonic *Ccl2* expression (Figure 7A and Figure 8C,D). On the other hand, the abundance of *Firmicutes* and *Faecalibaculum*, which was decreased by AC supplementation during early life, was positively correlated with intestinal *Igf1* and *Igf1r* expression, respectively (Figure 7B,C).

While it is accurate that other compounds exhibiting similar properties may elicit analogous effects, the distinctiveness of AC lies in its unique amalgamation of bioactive compounds. The synergistic interplay among these compounds may constitute the pivotal factor contributing to its efficacy, with potential additional benefits or mechanisms that distinguish it from other substances. In the context of intestinal cancer, AC demonstrates anti-inflammatory and anti-cancer effects. This study reveals that AC not only alleviates insulin resistance but also ameliorates dysbiosis induced by a HF diet. This is achieved through the augmentation of lean-associated bacteria and SCFA-producing bacteria, coupled with the reduction of obesity-associated bacteria, all of which are intricately linked to obesity and insulin resistance. Furthermore, the advantages of early-life AC consumption extend to mitigating metabolic dysregulation and gut dysbiosis. The anti-cancer and anti-inflammatory effects persist into later stages of life, even after transitioning to standard lifecycle maintenance diets. These findings underscore the profound influence of microbiota composition on the development of CRC, with early-life AC consumption significantly impacting this relationship.

The study acknowledges certain limitations that merit consideration. Notably, the mouse model employed for inducing diet-related intestinal tumors is specific to the small intestine. While the tumorigenic mechanisms share similarities with those observed in humans, such as chronic inflammation, genetic mutations, insulin resistance, and dysbiosis, it is essential to recognize that the pathogenesis diverges to some extent.

Despite providing valuable insights into associations and certain mechanistic pathways, the preclinical animal study inherently offers a model-specific perspective. Therefore, there is a recognized imperative for additional in vitro studies to substantiate the molecular mechanisms identified. Furthermore, to enhance translational relevance, the initiation of clinical studies is warranted to validate the observed effectiveness in human contexts. These sequential steps will collectively enhance the robustness and applicability of the findings from this preclinical investigation.

Regarding future directions, the intricate relationship between early-life nutrition, obesity, and colorectal tumorigenesis prompts the recognition of its complexity. There may exist additional tumorigenic pathways related to poor early-life nutrition that merit thorough investigation. Through the exploration of potential early-life risk factors and a systematic integration of findings, a deeper understanding of how early-life nutrition and obesity impact colorectal tumorigenesis can be attained. Furthermore, the present study’s results provide new insights into the development of young-onset cancers, thereby informing the design of clinical trials or nutritional interventions targeting this specific population. Overall, this study holds promise, and its findings may be translated into health-promoting practices or strategies to address the escalating burden of cancer among young adults.

## 5. Conclusions

In summary, by using the *APC^1638N^* intestinal cancer model, we examined how early-life nutrition, equivalent to childhood and adolescence in humans, affects intestinal tumorigenesis later in life. Our findings demonstrated a noteworthy role of AC in inhibiting intestinal tumorigenesis, especially in female mice. The results also suggested that AC supplementation during early life can alleviate insulin resistance, IGF-1 signaling, and Wnt/β-catenin signaling. Moreover, it altered the microbiota composition, suppressed inflammatory responses, created a microenvironment tending to suppress intestinal tumorigenesis, and these effects extended later in life after switching to normal lifecycle maintenance diets.

## Data Availability

The data presented in this study are available from the corresponding author upon reasonable request.

## Supplementary Materials

The following supporting information can be downloaded at: https://www.mdpi.com/article/10.3390/nu16152408/s1”, Table S1: Formula of experimental diets; Table S2: Primers for IGF-1 signaling, Wnt/β-catenin signaling, and inflammatory mediator-related genes; Table S3: Comparisons of plasma inflammatory mediators among *APC^1638N^* mice later in life that were fed with a high-fat diet (HF) with or without AC supplementation or a low-fat diet (LF) with or without AC supplementation during early life; Table S4: Comparisons of intestinal inflammatory mediators among *APC^1638N^* mice later in life that were fed with a high-fat diet (HF) with or without AC supplementation or a low-fat diet (LF) with or without AC supplementation during early life; Table S5: Comparisons of intestinal inflammatory mediators between *APC^1638N^* mice later in life that were fed with AC supplementation or without AC supplementation during early life; Figure S1: Correlation matrix of gut microbiota and intestinal expressions of selected oncogenes, cytokines, and chemokines; Figure S2: Full Western blot for phospho-Akt; Figure S3: Full Western blot for phospho-Mek; Figure S4: Full Western blot for phospho-Erk1/2; Figure S5: Full Western blot for active β-catenin; Figure S6: Full Western blot for phospho-GSK-3β.
