# Supplementary material for: Antrodia camphorata Supplementation during Early Life Alters Gut Microbiota and Inhibits Young-Onset Intestinal Tumorigenesis in APC1638N Mice Later in Life
Source: Nutrients. 2024 Jul 25;16(15):2408. doi: 10.3390/nu16152408 (PMC11314251; doi:10.3390/nu16152408)
Supplement: Supplementary file 1 [file nutrients-16-02408-s001.zip › nutrients-3080573-supplementary.pdf]

Supplementary Materials

Antrodia Camphorata Supplementation during Early Life Alters Gut Microbiota and Inhibits Young-Onset Intestinal Tumorigenesis in APC^{1638N} Mice Later in Life

Tingchun Lin, Lauren Daddi, Ying Tang, Yanjiao Zhou, Buping Liu, Matthew D. Moore, Zhenhua Liu*

***Correspondence:**
Corresponding Author: Zhenhua Liu, Ph.D.
Email: zliu@nutrition.umass.edu; Tel.: +1-413-545-1075; Fax: +1-413-545-1074

Supplementary Tables

	Chow diet	Low-fat diet		High-fat diet	
	kcal %	kcal %		kcal %	
Protein	26	20		20	
Carbohydrate	60	70		20	
Fat	14	10		60	
Total	100	100		100	
Ingredient		gm	kcal	gm	kcal
Casein, 80 Mesh		200	800	200	800
L-Cystine		3	12	3	12
Corn Starch		315	1260	0	0
Maltodextrin 10		35	140	125	500
Sucrose		350	1400	68.8	275.2
Cellulose, BW200		50	0	50	0
Soybean Oil		25	225	25	225
Lard		20	180	245	2205
Mineral Mix S10026		10	0	10	0
DiCalcium Phosphate		13	0	13	0
Calcium Carbonate		5.5	0	5.5	0
Potassium Citrate, 1 H_2O		16.5	0	16.5	0
Vitamin Mix V10001		10	40	10	40
Choline Bitartrate		2	0	2	0
Total		1055	4057	773.8	4057

Supplementary Table S1. Formula of experimental diets

Pathway	Gene	Forward Primer	Reverse Primer
IGF-1 signaling	*Igf1*	CACATCATGTCGTCTTCACACC	GGAAGCAACACTCATCCACAATG
	Igf1r	TGACATCCGCAACGACTATCA	CCAGTGCGTAGTTGTAGAAGAGT
	Akt	CATGAACGACGTAGCCATTG	CCATCGTTCTTGAGGAGGAA
	Hdac6	TCCACCGGCCAAGATTCTTC	GCCTTTCTTCTTTACCTCCGCT
	c-Jun	GGGACACAGCTTTCACCCTA	GAAAAGTAGCCCCCAACCTC
Wnt/β-catenin signaling	*c-Myc*	TGAAGTTCACGTTGAGGGG	AGAGCTCCTCGAGCTGTTTG
	Ccnd1	GGGTGGGTTGGAAATGAAC	TCCTCTCCAAAATGCCAGAG
	Axin2	TGCATCTCTCTCTGGAGCTG	ACTGACCGACGATTCCATGT
Inflammatory mediators	*Tnf*	CAGGCGGTGCCTATGTCTC	CGATCACCCCGAAGTTCAGTAG
	Il1β	GAAATGCCACCTTTTGACAGTG	TGGATGCTCTCATCAGGACAG
	Il6	CTGCAAGAGACTTCCATCCAG	AGTGGTATAGACAGGTCTGTTGG
	Il17A	TCAGCGTGTCCAAACACTGAG	CGCCAAGGGAGTTAAAGACTT
	Ccl2	TAAAAACCTGGATCGGAACCAAA	GCATTAGCTTCAGATTTACGGGT
	Ptgs2	TTCCAATCCATGTCAAAACCGT	AGTCCGGGTACAGTCACACTT
	Tgfb1	CCACCTGCAAGACCATCGAC	CTGGCGAGCCTTAGTTTGGAC
Reference	*Gapdh*	AGGTCGGTGTGAACGGATTTG	GGGGTCGTTGATGGCAACA

Supplementary Table S2. Primers for IGF-1 signaling, Wnt/β-catenin signaling, and inflammatory mediators related genes

(pg/mL)	LF diet	LF diet + AC	HF diet	HF diet + AC
TNF-α	51.060 ± 6.593	53.217 ± 6.511	43.060 ± 5.297	55.627 ± 6.519
IL-6	0.459 ± 0.095	0.249 ± 0.060 #	0.198 ± 0.049	0.305 ± 0.074
IL-17A	15.597 ± 4.278	8.816 ± 1.330	17.203 ± 2.927	21.338 ± 2.889
CCL2	0.427 ± 0.062	0.442 ± 0.048	0.382 ± 0.034	0.396 ± 0.041

Female (pg/mL)	LF diet	LF diet + AC	HF diet	HF diet + AC
TNF-α	40.140 ± 5.058	46.160 ± 3.827	36.667 ± 5.514	46.857 ± 7.550
IL-6	0.430 ± 0.143	0.188 ± 0.053	0.192 ± 0.084	0.324 ± 0.118
IL-17A	11.614 ± 1.867	8.516 ± 1.249	18.600 ± 2.652	17.379 ± 2.819
CCL2	0.483 ± 0.099	0.465 ± 0.078	0.408 ± 0.058	0.336 ± 0.040

Male (pg/mL)	LF diet	LF diet + AC	HF diet	HF diet + AC
TNF-α	61.980 ± 10.507	58.257 ± 10.774	52.650 ± 9.128	70.975 ± 8.166
IL-6	0.488 ± 0.140	0.351 ± 0.128	0.205 ± 0.052	0.276 ± 0.075
IL-17A	19.580 ± 8.421	9.030 ± 2.190	15.526 ± 5.941	26.880 ± 5.023
CCL2	0.371 ± 0.075	0.403 ± 0.019	0.351 ± 0.027	0.480 ± 0.070

Supplementary Table S3. Comparisons of plasma inflammatory mediators among APC^{1638N} mice later in life that were fed with a high-fat diet (HF) with or without AC supplementation or a low-fat diet (LF) with or without AC supplementation during early life. Plasma samples were collected on week 24. Data was presented as mean ± SEM, n=10-12 for each group. $^{\#}p < 0.1$, as diet with AC supplementation compared to diet without AC supplementation. Red color specifies the statistical significance as the comparison between HF and LF.

(pg/mL)	LF diet	LF diet + AC	HF diet	HF diet + AC
TNF-α	1.332 ± 0.451	0.411 ± 0.048 #	2.504 ± 1.061	0.555 ± 0.130
IL-6	45.002 ± 10.919	22.343 ± 3.082 *	38.977 ± 13.069	25.444 ± 2.852
IL-17A	3.871 ± 0.641	3.049 ± 0.238	3.805 ± 0.950	2.609 ± 0.243
CCL2	0.499 ± 0.081	0.473 ± 0.054	0.383 ± 0.043	0.393 ± 0.043

Female (pg/mL)	LF diet	LF diet + AC	HF diet	HF diet + AC
TNF-α	1.032 ± 0.459	0.466 ± 0.057	2.292 ± 1.241	0.451 ± 0.160
IL-6	46.786 ± 17.227	23.146 ± 2.755	44.883 ± 20.896	25.293 ± 4.180
IL-17A	3.758 ± 0.652	2.910 ± 0.470	3.417 ± 0.862	2.606 ± 0.377
CCL2	0.517 ± 0.128	0.375 ± 0.084	0.328 ± 0.031	0.309 ± 0.035

Male (pg/mL)	LF diet	LF diet + AC	HF diet	HF diet + AC
TNF-α	1.831 ± 0.999	0.377 ± 0.068 #	2.856 ± 2.299	0.814 ± 0.077
IL-6	43.219 ± 15.430	21.770 ± 5.110	31.891 ± 16.167	25.654 ± 4.120
IL-17A	3.984 ± 1.191	3.148 ± 0.262	4.271 ± 1.937	2.614 ± 0.302
CCL2	0.481 ± 0.113	0.555 ± 0.057	0.450 ± 0.082	0.510 ± 0.062

Supplementary Table S4. Comparisons of intestinal inflammatory mediators among *APC*^{1638N} mice later in life that were fed with a high-fat diet (HF) with or without AC supplementation or a low-fat diet (LF) with or without AC supplementation during early life. Intestine tissues were collected on week 24. Data was presented as mean ± SEM, n=10-12 for each group. $^{\#}p < 0.1$, $^{*}p < 0.05$, as diet with AC supplementation compared to diet without AC supplementation. Red color specifies the statistical significance as the comparison between HF and LF.

(pg/mL)	Diet without AC	Diet with AC
TNF-α	1.918 ± 0.577	0.478 ± 0.066 *
IL-6	41.846 ± 8.410	23.893 ± 2.079 *
IL-17A	3.837 ± 0.570	2.829 ± 0.172 #
CCL2	0.438 ± 0.045	0.431 ± 0.035

Female (pg/mL)	Diet without AC	Diet with AC
TNF-α	1.662 ± 0.658	0.457 ± 0.097
IL-6	45.748 ± 13.158	24.398 ± 2.610
IL-17A	3.572 ± 0.532	2.733 ± 0.284
CCL2	0.414 ± 0.064	0.336 ± 0.039

Male (pg/mL)	Diet without AC	Diet with AC
TNF-α	2.344 ± 1.144	0.502 ± 0.095
IL-6	37.555 ± 10.703	23.388 ± 3.349
IL-17A	4.128 ± 1.073	2.925 ± 0.205
CCL2	0.465 ± 0.066	0.534 ± 0.041

Supplementary Table S5. Comparisons of intestinal inflammatory mediators between APC^{1638N} mice later in life that were fed with AC supplementation or without AC supplementation during early life. Intestine tissues were collected on week 24. Data was presented as mean ± SEM, n=21-24 for each group. $^{\#}p < 0.1$, $^{*}p < 0.05$, as compared to diet without AC supplementation.

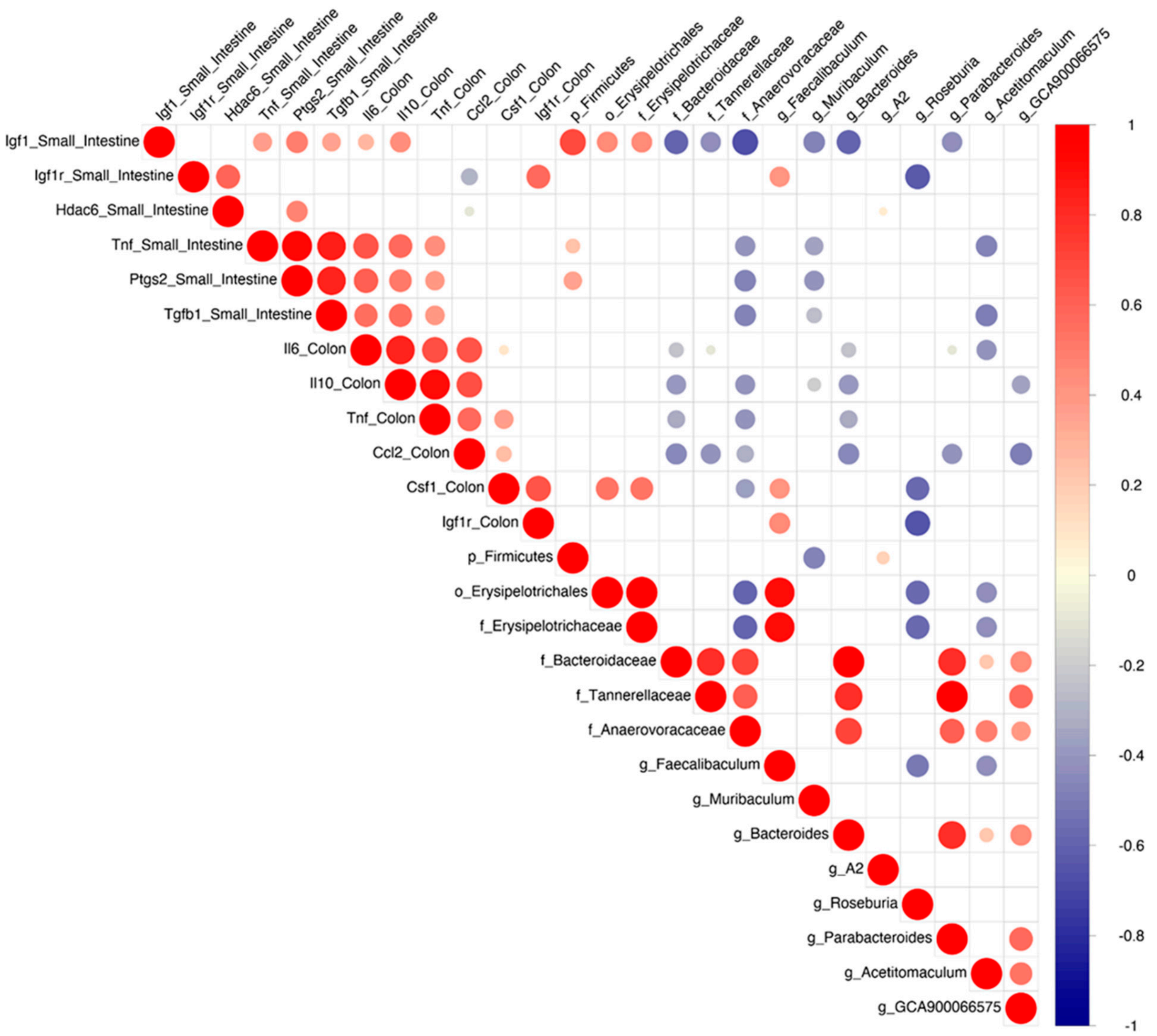

Supplementary Figure S1. Correlation matrix of gut microbiota and intestinal expressions of selected oncogenes, cytokines and chemokines. Blue or red dot represents a significant coefficient.

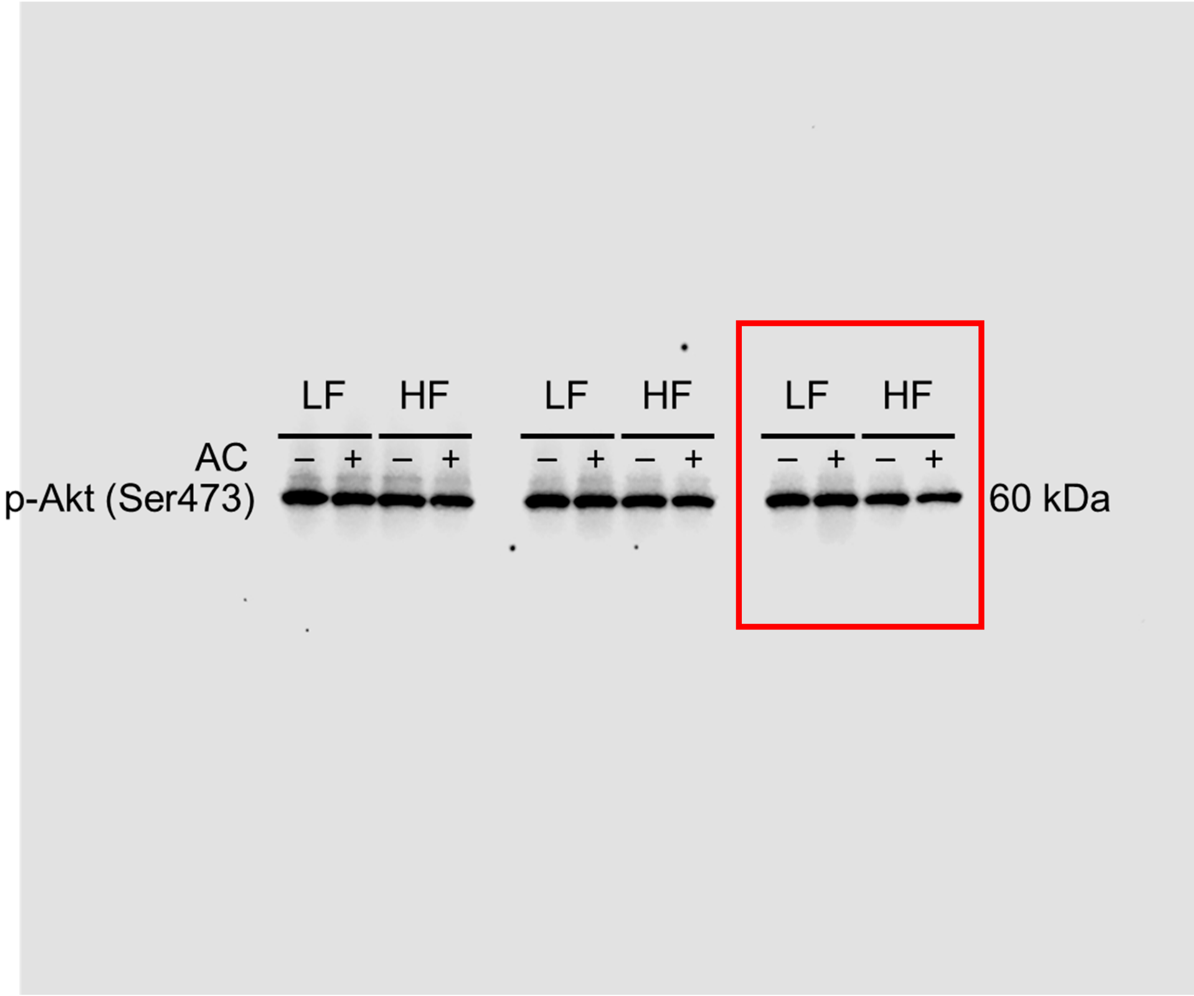

Supplementary Figure S2. Full western blot for phospho-Akt.

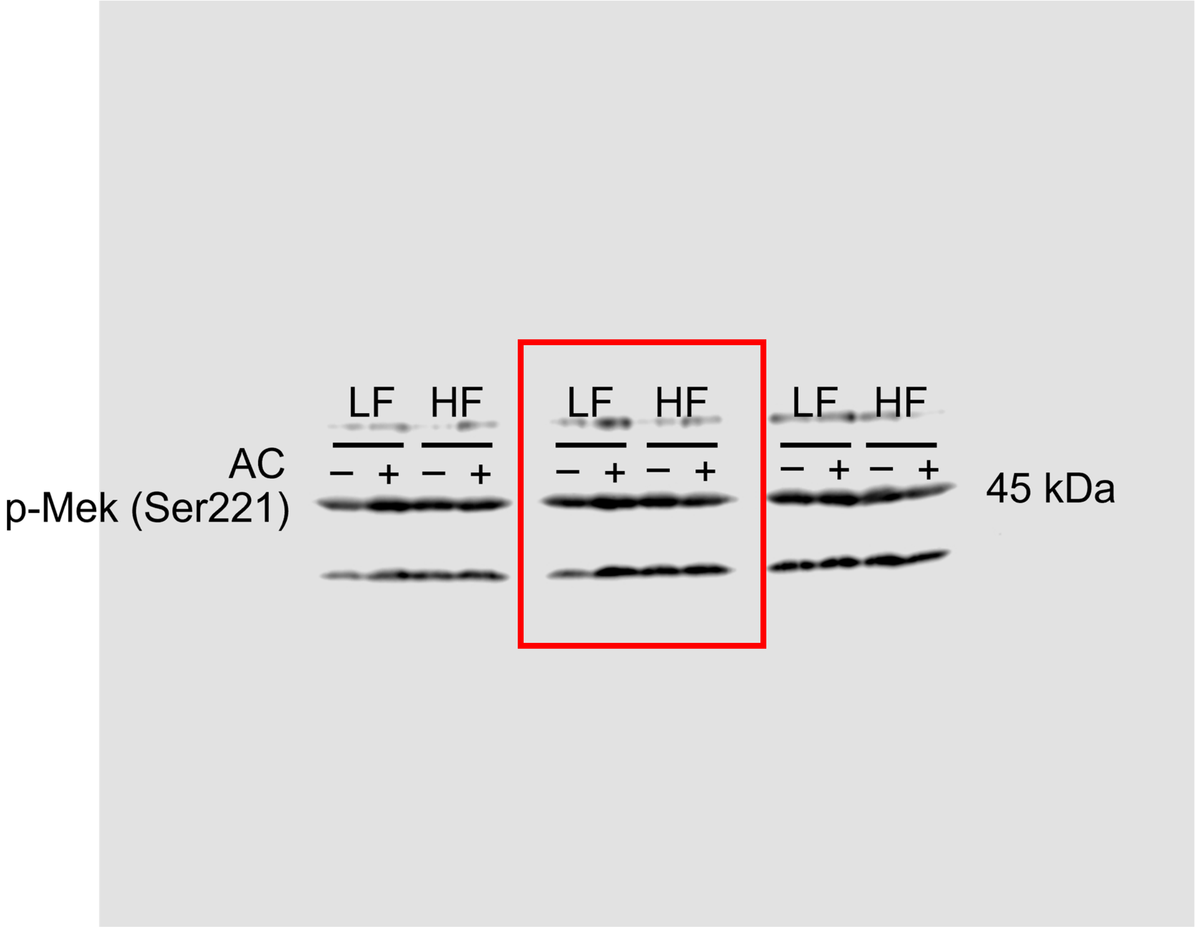

Supplementary Figure S3. Full western blot for phospho-Mek.

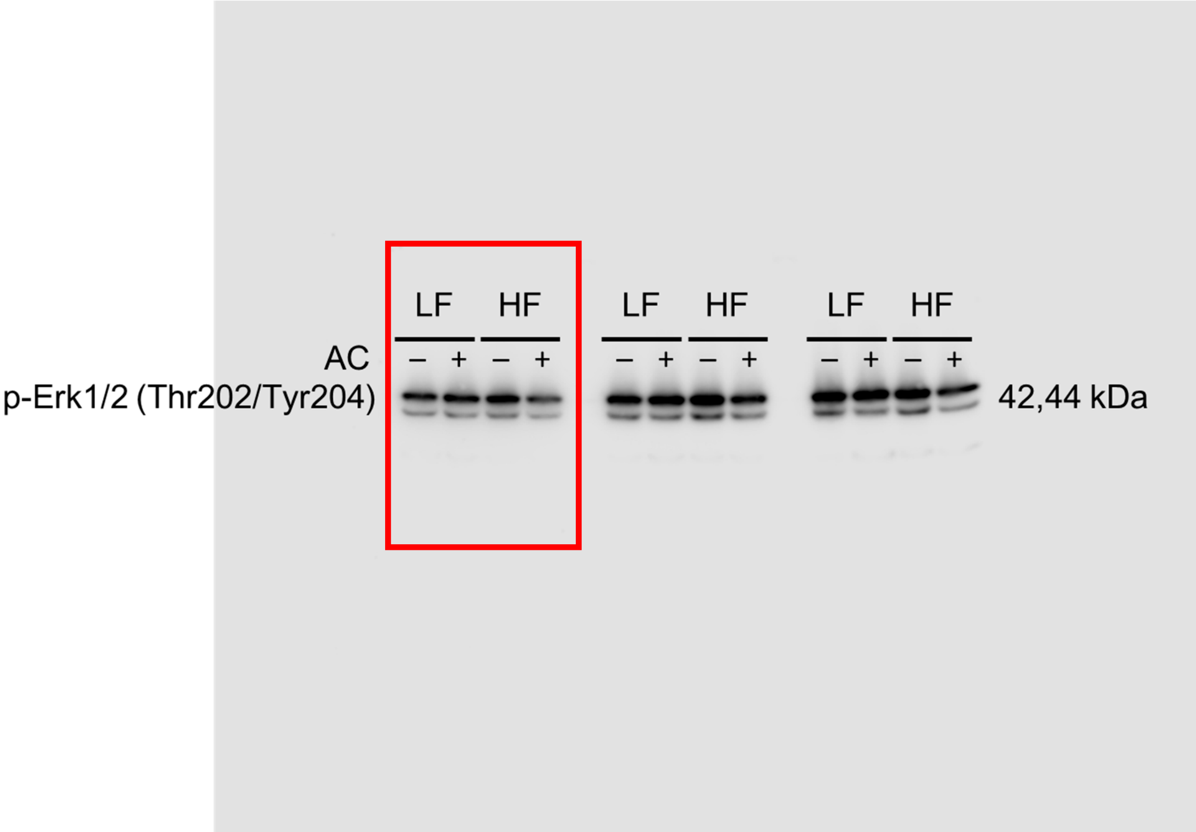

Supplementary Figure S4. Full western blot for phospho-Erk1/2.

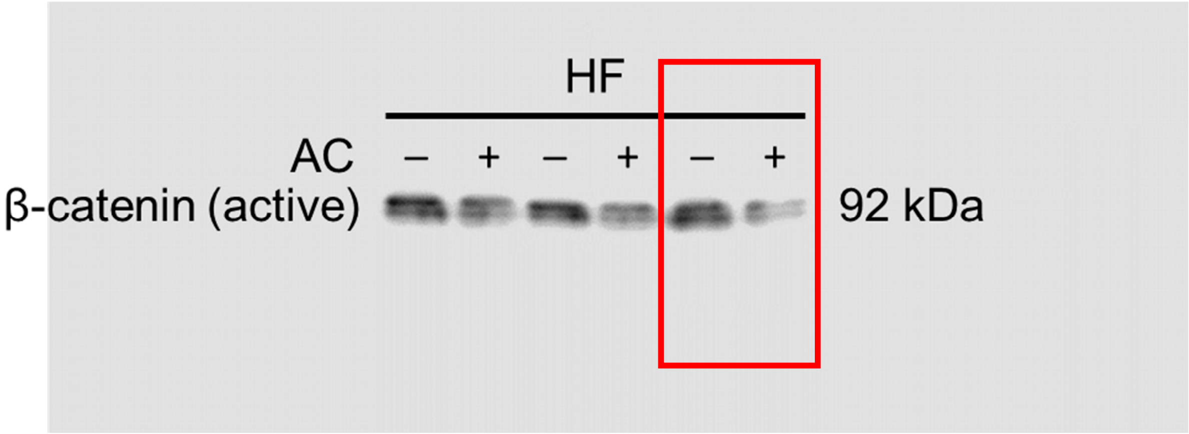

Supplementary Figure S5. Full western blot for active β-catenin.

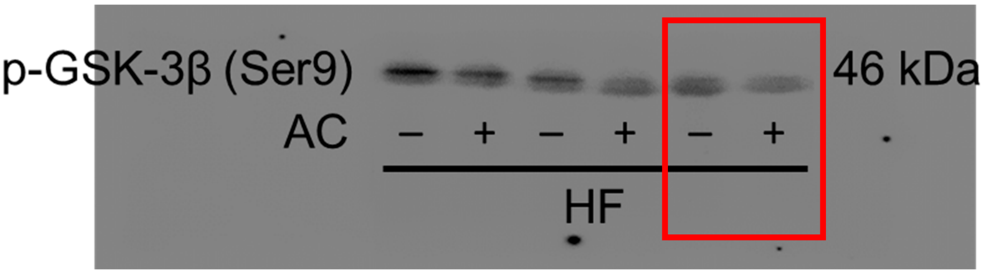

Supplementary Figure S6. Full western blot for phospho-GSK-3β.
